# Investigations into the impact of non-coding RNA on the sensitivity of gastric cancer to radiotherapy

**DOI:** 10.3389/fphys.2023.1149821

**Published:** 2023-02-24

**Authors:** Muhammad Usman, Aferin Beilerli, Albert Sufianov, Valentin Kudryashov, Tatiana Ilyasova, Pavel Balaev, Andrei Danilov, Hong Lu, Ilgiz Gareev

**Affiliations:** ^1^ Department of Medical Imaging, Central Hospital Affiliated to Chongqing University of Technology, Chongqing, China; ^2^ Department of Obstetrics and Gynecology, Tyumen State Medical University, Tyumen, Russia; ^3^ Department of Neurosurgery, Sechenov First Moscow State Medical University (Sechenov University), Moscow, Russia; ^4^ Department of Internal Diseases, Bashkir State Medical University, Ufa, Russia; ^5^ Gastric Cancer Center, West China Hospital of Sichuan University, Chengdu, Sichuan, China; ^6^ Department of Oncology and Radiology, Ural State Medical University, Yekaterinburg, Russia; ^7^ Department of Clinical Pharmacology, Smolensk State Medical University, Smolensk, Russia; ^8^ Educational and Scientific Institute of Neurosurgery, Рeoples’ Friendship University of Russia (RUDN University), Moscow, Russia

**Keywords:** non-coding RNA, radiotherapy, oncogenesis, therapeutic targets, biomarkers, gastric cancer, biofluids

## Abstract

Non-coding RNAs (ncRNAs) are a newly discovered functional RNA different from messenger RNA, which can participate in regulating the occurrence and development of tumors. More and more research results show that ncRNAs can participate in the regulation of gastric cancer (GC) radiotherapy response, and its mechanism may be related to its effect on DNA damage repair, gastric cancer cell stemness, cell apoptosis, activation of epidermal growth factor receptor signaling pathway, etc. This article summarizes the relevant mechanisms of ncRNAs regulating the response to radiotherapy in gastric cancer, which will be directly important for the introduction of ncRNAs particularly microRNAs (miRNAs), long non-coding RNAs (lncRNAs) and circular RNAs (circRNAs) into clinical medicine as biomarkers and therapeutic targets.

## 1 Introduction

**FIGURE 1:**
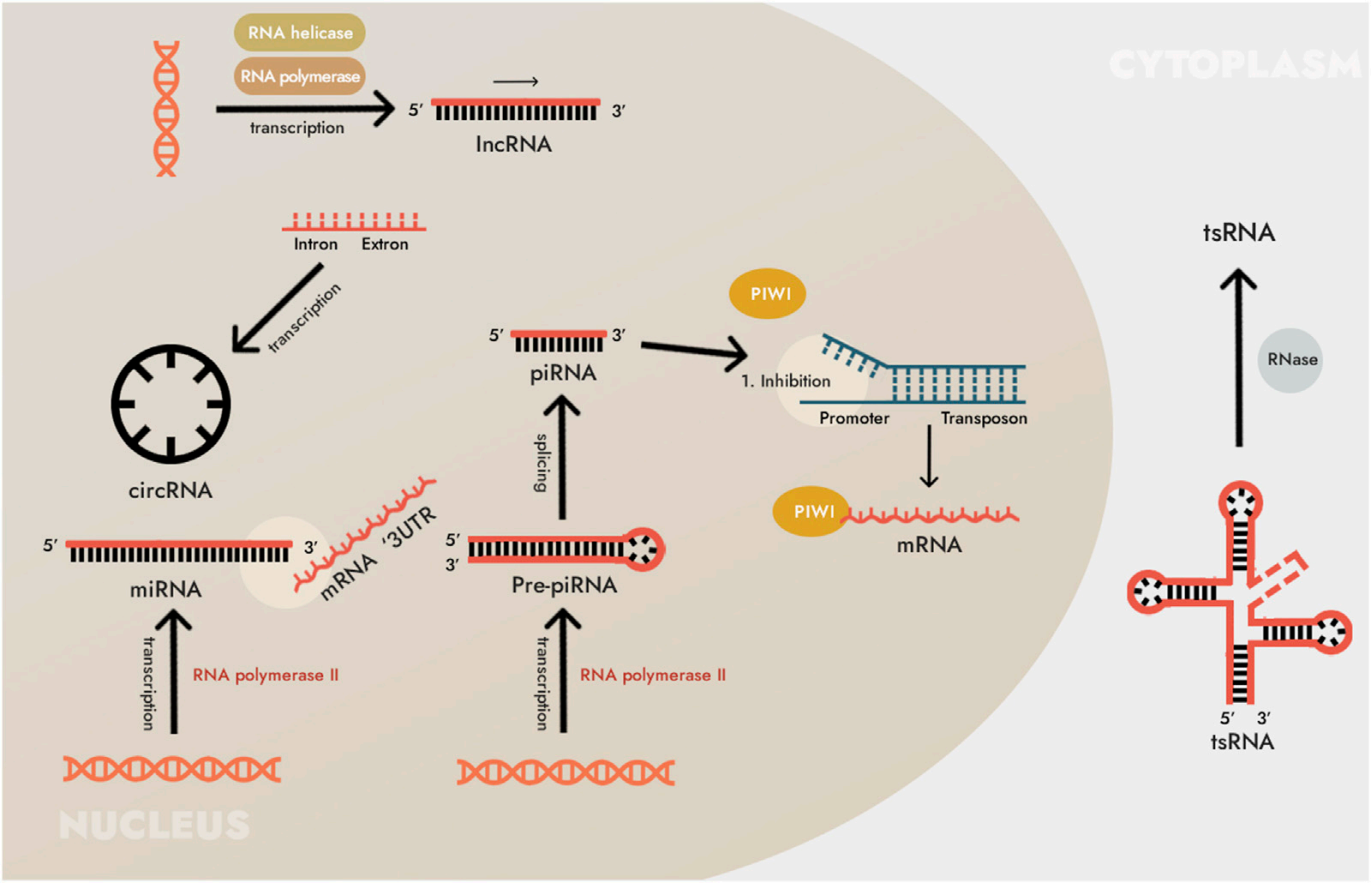
Biosynthesis of non-coding RNAs (ncRNAs). The main types of ncRNAs are microRNAs (miRNAs), long non-coding RNAs (lncRNAs) and circular RNAs (circRNAs). In addition to those listed, many other types of RNA are also included in ncRNAs. For instance, small ncRNAs include RNAs that interact with PIWI proteins (piRNA, piwi-interacting RNA, piwiRNA), transfer RNA (tRNA)-derived small non-coding RNA (tsRNA), and others. However, these types of ncRNAs have not been found in human cells, and/or their pathogenetic and diagnostic (as well as therapeutic) significance has not yet been shown, so we do not consider them here.

The incidence of gastric cancer (GC) ranks fifth in the world, and its death rate ranks third in the world (Smyth et al., 2020). Because the early symptoms of GC are often hidden and atypical, many patients usually present with advanced disease when they see a doctor. Radiation therapy (radiotherapy), as one of the main treatment methods for GC, has shown the advantages of reducing the recurrence rate and prolonging the survival of patients (Leong, 2005; Foo et al., 2014). However, due to the low sensitivity of GC to radiotherapy, an important problem remains the resistance of this tumor and, in particular, what mechanisms are involved in this (Pasechnikov et al., 2014; Ruan et al., 2020). Therefore, it is necessary to develop tumor-targeted drugs or radiosensitizers to enhance the radiosensitivity of GC and improve the radiotherapy efficacy of GC patients. Studies have found that the aberrant expression of non-coding RNAs (ncRNAs) are involved in regulating the radiotherapy sensitivity of various tumors such as nasopharyngeal carcinoma (NPC), non-small cell lung cancer (NSCLC), colorectal cancer (CC), GC and significantly affects the radiotherapy efficacy of tumors (Slack and Chinnaiyan, 2019; Gareev et al., 2020; Machlowska et al., 2020; Yan and Bu, 2021). NcRNAs are usually divided into basic structure type and regulatory type according to different functions. Regulatory ncRNAs are mainly composed of long non-coding RNAs (lncRNAs), microRNAs (miRNAs) and circular RNAs (circRNAs) (Beylerli et al., 2021) (Figure 1). This article reviews the dysregulation of ncRNAs in GC, summarizes and analyzes the research results of ncRNAs related to GC radiotherapy sensitivity, and explores new directions for improving the prognosis of GC patients after radiotherapy.

## 2 Regulation of DNA damage by ncRNAs in GC cells

Damage to DNA and cell membranes is considered to be the main cause of radiation-induced cancer cell death (Beylerli et al., 2021). Normal and cancer cells have the ability to sense DNA damage and initiate DNA damage repair, the DNA damage response. The DNA damage response plays an important role in sensing DNA double-strand breaks, inducing cell cycle arrest, and initiating DNA repair (Wang and Xie, 2022). The DNA damage response manifests as a signaling cascade in which DNA-damaging factors first activate telangiectatic ataxia mutant factors, which subsequently activate cellular checkpoint kinases, phosphorylate histone 2A variants, and inhibit cell entry into S and M phases, leading to cell cycle arrest and initiation of damage repair to maintain genome stability. The study by Hu et al. confirmed that, under X-ray irradiation, hsa-let-7 g can significantly increase the radiosensitivity of GC by reducing the expression of telangiectasia ataxia mutation factor in GC cells and indirectly inhibiting the activation of DNA damage response (Hu et al., 2015).

DNA double-strand breaks are the most prevalent and potent type of damage induced by radiation therapy. The role of ncRNA in DNA double-strand damage repair has been confirmed (Nickoloff et al., 2020). In SNU-638 GC cells, ectopically expressed miR-196b can reduce the expression of DNA repair protein RAD23B, leading to the blockage of DNA damage repair mechanism in GC cells, inducing cell death, and increasing the radiosensitivity of GC cells (Tsai et al., 2010). Tissue-based GC-related ncRNAs are listed in Table 1, focusing particularly on their involvement in DNA damage (Qin et al., 2018; Huang et al., 2019; Manoel-Caetano et al., 2019; Zhang et al., 2019; Zhang et al., 2020; Guo et al., 2022; Gupta et al., 2022)

**TABLE 1:** Some non-coding RNAs (ncRNAs) that regulate DNA damage in gastric cancer (GC) cells under the influence of radiotherapy.

NcRNA	Model study	Expression	Target gene/pathway	Type of ncRNA	Biological function	References
miR-21, miR-24 and miR-421	In silico and *in vitro*	Up	ATM/ATR/H2AX	Oncogene	Recognition and repair of DNA damage	Manoel-Caetano et al. (2019)
miR-192 and miR-215	*In vivo* and *in vitro*	Up	SET8 and p53	Oncogene	Promotes DNA damage repair and promotes progression of GC	Zhang et al. (2020)
miR-129-3p	*In vitro*	Down	SUMO-activating enzyme subunit 1 (SAE1)	Tumor suppressor	Induces more DNA damage and cell apoptosis, and inhibits GC cell proliferation, migration and invasion	Zhang et al. (2019)
lncRNA GAS5	Mathematical model	Down	ATM/p38 MAPK and miR-34c	Tumor suppressor	Stimulate of DNA damage and enhances the radiosensitivity of GC	Gupta et al. (2022)
lncRNA FOXD2-AS1	*In vitro*	Up	SETD1A	Oncogene	Promotes DNA damage repair and reduces the radiosensitivity of GC	Guo et al. (2022)
lncRNA MDC1-AS	*In vivo* and *in vitro*	Up	MDC1	Oncogene	Promotes DNA damage repair and significantly inhibits cell proliferation and metastasis	Qin et al. (2018)
Circular RNA AKT3	*In vivo* and *in vitro*	Up	PIK3R1	Oncogene	Promotes DNA damage repair and inhibits the apoptosis of GC cells	Huang et al. (2019)

Abbreviations: ATM, Ataxia-telangiectasia mutated; ATR, Ataxia telangiectasia and Rad3-related protein; SAE1, SUMO-activating enzyme subunit one; MAPK, Mitogen-activated protein kinase; MDC, Macrophage-derived/CCL22 chemokine; PIK3R1, Phosphatidylinositol 3-kinase regulatory subunit alpha; GAS5, Growth arrest-specific five; FOXD2-AS1, Forkhead box D2 adjacent apposite strand RNA, one; MDC1-AS, Mediator of DNA, damage checkpoint protein one; AKT3, AKT, serine/threonine kinase 3.

## 3 Regulation of ncRNA on tumor cell stemness in GC

It's no secret that, cancer stem cells are more resistant to radiation than mature cancer cells. Studies have shown that cancer stem cells have a strong ability to scavenge or reduce the level of reactive oxygen species (ROS), resulting in less DNA damage than mature cancer cells (Gareev et al., 2021). Currently, the Wnt/β-catenin pathway is considered to be one of the main targets of anti-tumor stem cell therapy. Studies have found that lncRNA HNF1A antisense RNA 1 (HNF1A-AS1), miR-501-5, and circFAM73A can promote the stemness of GC cells by activating the downstream Wnt/β-catenin pathway (Fan et al., 2016; Liu et al., 2018; Xia et al., 2021).

In addition, lncRNA metastasis-associated lung adenocarcinoma transcript 1 (MALAT1) promotes the stemness of GC cells by combining with sex determining region Y (SRY)-related HMG-box 2 (SOX) mRNA, Sox2 SRY (sex determining region Y)-box 2 mRNA, and knockdown of lncRNA MALAT1 can enhance the radiosensitivity of GC cells (Xu et al., 2021). In summary, some ncRNAs may be potential targets for GC radiotherapy by promoting the stem cell-like characteristics of GC, enhancing the scavenging ability of reactive ROS, reducing the damage effect of radiation on GC cells, and reducing the radiosensitivity of GC. A variety of ncRNAs have been reported to be involved in tumor cell stemness in GC (Table 2) (Song et al., 2018; Hong et al., 2019; Song et al., 2019; Sun et al., 2019; Zhao et al., 2020; Ni et al., 2021; Taghehchian et al., 2022; Xing et al., 2022; Zhang et al., 2022; Liu et al., 2023).

**TABLE 2:** Some non-coding RNAs (ncRNAs) that regulate tumor cell stemness in gastric cancer (GC) under the influence of radiotherapy.

NcRNA	Model study	Expression	Target gene/pathway	Type of ncRNA	Biological function	References
miR-375	*In vivo* and *in vitro*	Down	SLC7A11	Tumor suppressor	Inhibits the stemness and can induce ferroptosis of GC cells	Ni et al. (2021)
miR-18	*In vitro*	Up	Meis2 and HMGB3	Oncogene	Promotes the stemness of GC cells	Zhang et al. (2022)
miR-216a-3p	*In vitro*	Down	BRD4/Wnt/β-catenin pathway	Tumor suppressor	Promotes the stemness of GC cells	Song et al. (2019)
lncRNA PTCSC3	*In vitro*	Down	PTCSC3	Tumor suppressor	Inhibits the stemness and GC cells proliferation	Hong et al. (2019)
lncRNA THOR	*In vitro*	Down	SOX9	Tumor suppressor	Inhibits the stemness	Song et al. (2018)
lncRNA LOXL1-AS1	*In vitro*	Up	miR‐708‐5p and USF1	Oncogene	Promotes the stemness and contributes to GC cells proliferation, migration and EMT. Reflects poor prognosis	Sun et al. (2019)
lncRNA LINC00332	In silico, *in vitro* and bioinformatics	Down	MMP-13	Tumor suppressor	Inhibits the stemness and proliferation, migration, and invasion of GC cells	Taghehchian et al. (2022)
circ-NOTCH1	*In vivo* and *in vitro*	Up	miR-449c-5p/MYC/NOTCH1 axis	Oncogene	Promotes metastasis and stemness in GC	Zhao et al. (2020)
circRPPH1	*In vitro*	Up	SLC7A11	Oncogene	Promotes the stemness and can regulate ferroptosis of GC cells	Liu et al. (2023)
circ0007360	*In vivo* and *in vitro*	Up	miR-762/IRF7 axis	Tumor suppressor	Inhibitory effects of circ0007260 on the survival, migration, invasion, and stemness of GC cells	Xing et al. (2022)

Abbreviations: PTCSC3, Papillary thyroid carcinoma susceptibility candidate three; LOXL1-AS1, LOXL1 Antisense RNA, one; LINC00332, Long intergenic non-protein coding RNA, 332; SLC7A11, Meis2, Meis homeobox two; HMGB3, High-mobility group protein B3; BRD4, Bromodomain-containing protein 4; PTCSC3, Papillary thyroid carcinoma susceptibility candidate three; SOX9, SRY-Box transcription factor 9; USF1, Upstream stimulatory factor 1; MMP-13, Matrix metalloproteinase 13; NOTCH1, Neurogenic locus notch homolog protein one; SLC7A11, Solute carrier family 7, membrane 11; IRF7, Interferon regulatory factor 7; EMT, Epithelial-mesenchymal transition.

## 4 Regulation of ncRNA on apoptosis of GC cells

When repair of DNA damage caused by radiation fails, cells initiate automatic death programs (apoptosis) to maintain genome stability. For radiation-induced cell damage, whether tumor cells choose to repair the damage or initiate apoptosis is of great significance to the prognosis of tumors.

The p53 gene is currently the most widely studied cell regulatory gene. Studies have shown that in p53-deficient GC cells, miR-34 can restore p53 function and induce cell apoptosis (Xiong et al., 2019). miR-375 can directly interact with the 3′-untranslated region (3′-UTR) mRNA of the p53 gene, negatively regulate p53 expression and downstream pathways, and reduce the radiosensitivity of GC cells by inhibiting apoptosis and causing cell cycle arrest (Xu et al., 2011). In addition, the study found that in GC cells after radiation exposure, inhibition of the miR-221/222 cluster can upregulate the expression of phosphatase and tensin homolog deleted on chromosome 10 (PTEN) in GC cells, activate phosphatidylinositol-3-hydroxykinase (PI3K)/Akt signaling pathway, induce apoptosis, and enhance the radiosensitivity of GC cells (Chun-Zhi et al., 2010). LncRNA growth arrest-specific 5 (GAS5) significantly inhibits GC cell proliferation, promotes apoptosis, and enhances radiosensitivity by targeting miR-196a (Li et al., 2016). It can be seen that the abnormal expression of ncRNA is closely related to the apoptosis of GC cells, which largely determines the sensitivity of GC cells to radiotherapy (Table 3) (He et al., 2014; Zhang et al., 2014; Mao et al., 2019; Wei et al., 2019; Liu et al., 2021a; Wang et al., 2021a; Qin et al., 2022; Wang et al., 2022; Zhou et al., 2022).

**TABLE 3:** Some non-coding RNAs (ncRNAs) that regulate stability to apoptosis in gastric cancer (GC) cells under the influence of radiotherapy.

NcRNA	Model study	Expression	Target gene/pathway	Type of ncRNA	Biological function	References
miR-4537	*In vitro*	Down	ZNF587	Tumor suppressor	Inhibits the ability of cell proliferation, but on the contrary, it promotes the ability of cell apoptosis and improves radiosensitivity of GC cells	Liu et al. (2021a)
miR-4766-5p	*In vitro*	Down	NKAP	Tumor suppressor	Induces GC cell apoptosis	Wei et al. (2019)
miR-300 and miR-642	*In vitro* and bioinformatics	Down	BCL2L11, GAS2, CASP8AP2, APAF1, DLC1, TP53, CASPS2, CASPS7, CASPS9, CASPS10, and BCL2L11	Tumor suppressor	Regulate cellular radiation response by modulating apoptosis and cell cycle regulation	He et al. (2014)
lncRNA LINC00152	*In vitro* and *in vivo*	Up	Bcl-2	Oncogene	Activates cell cycle signaling, promotes migration and invasion, and suppress apoptosis	Mao et al. (2019)
lncRNA OGFRP1	*In vitro*	Up	miR-149-5p/MAP3K3 axis	Oncogene	Promotes proliferation and suppresses GC cells radiosensitivity	Qin et al. (2022)
lncRNA CARLo-5	*In vitro*	Up	ERK/MAPK pathway	Oncogene	Promotes the GC cells proliferation and inhibits apoptosis	Zhang et al. (2014)
lncRNA SLC25A21-AS1	*In vitro*	Down	miR-15a-5p	Tumor suppressor	Inhibits cell malignant behaviors (e.g., promotes to apoptosis) and enhances cell radiosensitivity in GC	Wang et al. (2022)
circ_0003506	*In vitro* and *in vivo*	Up	miR-1256/BMPR2 axis	Oncogene	Downregulation of circ_0003506 inhibits radioresistance to repress proliferation, migration and invasion but increase apoptosis in radioresistant GC cells	Zhou et al. (2022)
circ_HN1	*In vitro* and *in vivo*	Up	miR-302b-3p/ROCK2 axis	Oncogene	Promotes tumor growth, cell proliferation, migration, invasion, and inhibit cell apoptosis in GC cells	Wang et al. (2021a)

Abbreviations: OGFRP1, Opioid growth factor receptor pseudogene one; SLC25A21-AS1, SLC25A21 antisense RNA, one; circ_HN1, circRNA, Jupiter microtubule associated homolog one; ZNF587, Zinc finger protein 587; NKAP, NF-kappa-B-activating protein; BCL2L11, recombinant human Bcl-2-like protein 11; GAS2, Growth arrest-specific protein two; CASP8AP2, Caspase eight associated protein two; APAF1, Apoptotic protease activating factor 1; DLC1, Deleted in liver cancer one; TP53, Tumor protein P53; CASPS2, Caspase two; CASPS7, Caspase seven; CASPS9, Caspase nine; CASPS10, Caspase 10; BCL2L11, Proapoptotic member of the B-cell CLL/lymphoma two; Bcl-2, B-cell lymphoma two MAP3K3, Mitogen-activated protein kinase kinase kinase three; ERK, Extracellular signal-regulated kinase; MAPK, Mitogen-activated protein kinase; BMPR2, Bone morphogenetic protein receptor type 2; ROCK2, Rho associated coiled-coil containing protein kinase two.

## 5 Activation of epidermal growth factor receptor (EGFR) signaling pathway by ncRNA

Radiation can cause abnormal expression of various genes in tumor cells, including epidermal growth factor receptor (EGFR). Overexpression of EGFR is related to lymphatic metastasis of GC, and can lead to growth and invasion of GC cells through the Akt pathway (Chen et al., 2021). As a stress response to radiation, EGFR is rapidly activated and induces the mitogen-activated protein kinase (MAPK)/extracellular signal-regulated kinase (ERK) and PI3K/Akt signaling pathways (Lei et al., 2022). Activation of these signaling pathways may repair radiation-induced DNA damage, evade apoptosis, and promote cell proliferation through homologous and non-homologous recombination (Molina-Castro et al., 2017).

**FIGURE 2:**
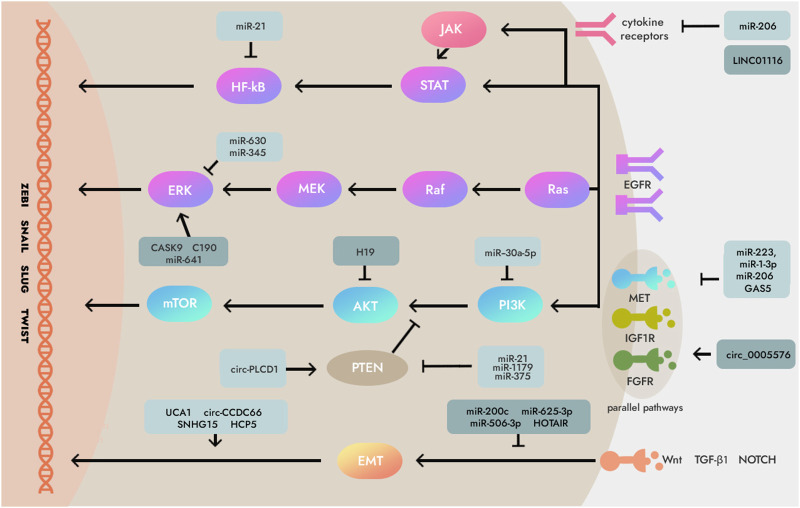
Mechanisms of non-coding RNAs (ncRNAs) which involved in essential signaling pathways downstream and parallel pathways of epidermal growth factor receptor (EGFR) in some human cancers including gastric cancer (GC). Several critical ncRNAs can regulate EGFR signaling pathways like Ras/Raf/MEK/ERK axis, PI3K/AKT/mTOR axis, JAK/STAT/NF‐κB, PTEN, and Wnt/TGF‐β1/NOTCH. Note: MEK, Mitogen-activated protein kinase; ERK, Extracellular signal-regulated kinase; PI3K, Phosphoinositide 3-kinases; AKT, Serine/threonine-protein kinase; mTOR, Mammalian target of rapamycin; JAK, Janus kinase two; STAT, Signal transducer and activator of transcription; NF‐κB, Nuclear factor kappa-light-chain-enhancer of activated B cells; NOTCH, Neurogenic locus notch homolog protein; EMT, epithelial‐mesenchymal transition; IGF1R, Insulin‐like growth factor 1 receptor; PTEN, Phosphatase and ten sin homolog deleted on chromosome 10; TGF‐β1, Transforming growth factor‐β1; FGFR, Fibroblast growth factor receptor.

Previous studies have shown that anti-EGFR-targeted therapy is an effective radiosensitizer for EGFR-overexpressing GC cells and xenografts. This radiosensitization is associated with inhibition of GC cell proliferation and promotion of apoptosis (Dragovich and Campen, 2009). Recent studies have shown that a variety of ncRNAs mediate the expression of EGFR in GC, so these ncRNAs can be used as a medium to target and regulate the expression of EGFR, and then promote the apoptosis of GC cells in the process of radiotherapy, thereby improving the sensitivity of GC to radiotherapy (Carlomagno et al., 2017; D'Souza et al., 2020; Kong et al., 2021; Ye et al., 2022; Lazăr et al., 2016) (Figure 2). Therefore, analyzing the regulatory mechanism of ncRNA on EGFR expression is a new direction worth exploring to improve the radiosensitivity of GC, and the EGFR inhibitor derived from this is expected to be a selective and effective radiosensitizer for GC.

## 6 Regulation of epithelial-mesenchymal transition (EMT) by ncRNAs

A phenotypic change in tumor cells that may result in enhanced tumor cell motility and invasiveness, increased metastatic potential, and radiotherapy resistance (Lu et al., 2022). LncRNA HOX transcript antisense RNA (HOTAIR) can bind to miR-331-3p and inhibit its function, leading to upregulation of human epidermal growth factor receptor 2 (HER2) expression, promoting epithelial-mesenchymal transition (EMT) through HER2/Akt/HSF-1/slug signaling pathway. This may be related to the radiotherapy resistance of GC cells (Wang et al., 2015; Abdi et al., 2020).

**FIGURE 3:**
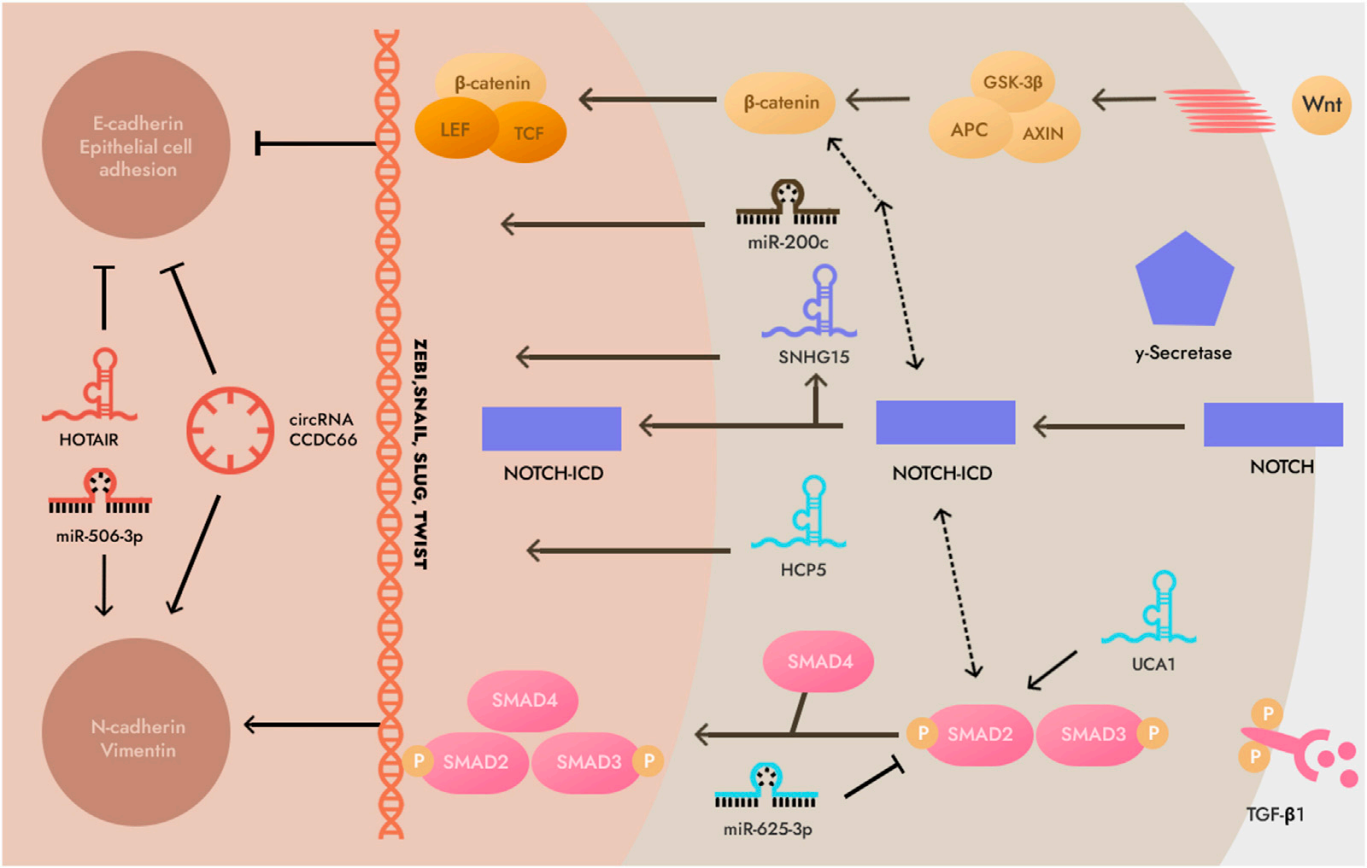
Non-coding RNAs (ncRNAs) involved in epithelial-mesenchymal transition (EMT). This illustration demonstrates that the EMT process is predominantly composed of transforming growth factor-β1 (TGF-β), WNT and neurogenic locus notch homolog protein (NOTCH) signaling pathways. Note: SMAD2,3,4, Mothers against decapentaplegic homolog 2,3,4; GSK3β, glycogen synthase kinase-3β; LEF, Lymphoid enhancer-binding factor; N-cadherin, Neural cadherin; TCF, T cell factor; NOTCH-ICD, intracellular domain of the NOTCH receptor.

MiR-544a-5p can act on cadherin E and Wnt/β-catenin to induce GC cell EMT through two independent pathways, and lncRNA RP11-789C1.1 inhibits EMT in GC *via* the RP11-789C1.1/miR-5003/cadherin E axis (Chen et al., 2018). LncRNA-h19 can combine with miR-141-5p to promote the EMT process of GC cells by up-regulating the expression of zinc finger E-box binding homeobox 1 (ZEB1) (Liu et al., 2022). Therefore, there may be a special signaling pathway between ncRNA and EMT, and through the regulation of EMT expression, it can affect the curative effect of GC cell radiotherapy (Figure 3) (Yang et al., 2015; Feng et al., 2019; Liu et al., 2021b; Beilerli et al., 2022).

## 7 Regulation of oxygen adaptive metabolism by ncRNAs

The most striking feature of tumor cells is the ability to metabolize energy by glycolysis even in the presence of sufficient oxygen, which is known as the Warburg effect. Studies have shown that aerobic glycolysis in malignant tumors is closely related to tumor radiotherapy resistance (Yuan et al., 2022). ROS play an important role in radiation-induced DNA damage. The generation of reactive oxygen species is mainly in the process of oxidative phosphorylation in cells. Electron leakage during mitochondrial electron transport is the main source of electrons for the generation of intracellular ROS, and glycolysis leads to reduced generation of ROS, which seriously affects ROS-induced radiation damage (Tanprasert et al., 2022).

It is evident from many recent studies that when NSCLC, cervical cancer (CC), and glioma cells are exposed to radiation, various ncRNAs such as miR-449a and lncRNA urothelial carcinoma-associated 1 (UCA1) in the cells target multiple functions in the glycolytic metabolic pathway, specifically by decreasing the rate of a key rate-limiting enzyme, leading to an increase in the sensitivity of tumor cells to radiation (Yao et al., 2015; Nie et al., 2016; Wang et al., 2019). This phenomenon also occurs in GC cells, indicating that ncRNAs may play a role in altering the metabolic mode of GC and influencing its radiation sensitivity. MiR-4290 inhibits pyruvate dehydrogenase kinase 1 (PDK1), inhibiting glycolysis (Qian et al., 2021). MiR-7 can inhibit the glycolysis, cell proliferation and colony formation of GC cells by regulating the expression of lactate dehydrogenase (Xie et al., 2014). Therefore, ncRNA may enhance the curative effect of GC radiotherapy by regulating the glycolysis process of GC cells, changing the metabolic mode of cells, and increasing the level of ROS in cells.

Hypoxia is a pathophysiological feature of solid malignancies. Under hypoxic conditions, hypoxia-inducible factor-1α (HIF-1α) is upregulated, activating hypoxic adaptation pathways, including angiogenesis, erythropoiesis, and glycolysis (Li et al., 2019). HIF-1α protects blood vessels after radiotherapy and regulates glycolysis and pentose phosphate pathways, which increases the antioxidant capacity of tumors, thereby counteracting the oxidative stress caused by radiotherapy and affecting the radiosensitivity of tumors (Yasui et al., 2008). Previous studies have found that ncRNAs can regulate tumor glycolysis by regulating HIF-1α and its downstream glycolysis-related enzymes, thereby affecting tumor radiosensitivity (Zheng et al., 2021; Xu et al., 2022). The high expression of lncRNA ZNFX1 antisense RNA 1 (ZFAS1) in gastric cardia adenocarcinoma (GCA) assists EPAS1 to enhance the epigenetic silencing of HIF-1α and promote the proliferation and metastasis of cancer cells (Zhu et al., 2020). MiR-376a binds lncRNA NUTM2A Antisense RNA 1 (NUTM2A-AS1) and negatively regulates HIF-1α to inhibit the invasion of GC (Wang et al., 2020). Therefore, the ncRNA/HIF-1α/glycolysis-related enzyme signaling pathway may be a potential target for regulating the radiosensitivity of GC, and it may become a new direction to improve the radiosensitivity of GC by regulating tumor glucose metabolism and tumor microenvironment. The ncRNAs that have been associated with the oxygen adaptive metabolism and their molecular pathways are listed in Table 4 (Tu et al., 2014; Hong et al., 2016; Fang et al., 2020; Wang et al., 2021b; Deng et al., 2021; Sun et al., 2021; Li et al., 2022; Tan et al., 2022; Yang et al., 2022).

**TABLE 4:** Some non-coding RNAs (ncRNAs) that regulate oxygen adaptive metabolism in gastric cancer (GC) cells.

NcRNA	Model study	Expression	Target gene/pathway	Type of ncRNA	Biological function	References
miR-21	*In vitro*	Up	PDCD4	Oncogene	Participates in balance of oxidation and antioxidant system in patients with GC	Tu et al. (2014)
miR-622	Bioinformatics, *in vitro* and *in vivo*	Down	NUAK1/p-protein kinase B (Akt) axis	Tumor suppressor	Decreases GC cell proliferation and migration but increases oxidative stress and inhibits the development of tumor	Yang et al. (2022)
miR-448	*In vitro*	Up	KDM2B	Oncogene	Promotes glycolytic metabolism of GC. Significantly associated with poor clinical outcomes of GC patients	Hong et al. (2016)
THUMPD3-AS1	*In vitro*	Down	miR-1252-3p and CXCL17	Tumor suppressor	Inhibits proliferation, migration, invasion and ROS accumulation of GC cells	Tan et al. (2022)
lncRNA LINC00242	*In vitro* and *in vivo*	Up	miR-1-3p/G6PD axis	Oncogene	Promotes cell proliferation and aerobic glycolysis and relieve the tumorigenesis	Deng et al. (2021)
lncRNA H19	Bioinformatics, *in vitro* and *in vivo*	Up	miR-519d-3p/LDHA axis	Oncogene	Promotes aerobic glycolysis, proliferation, and immune escape of GC cells	Sun et al. (2021)
circSLAMF6	*In vitro* and *in vivo*	Up	miR-204-5p/MYH9 axis	Oncogene	Promotes cell glycolysis, migration, and invasion of GC cells	Fang et al. (2020)
circDNMT1	*In vitro* and *in vivo*	Up	miR-576-3p/HIF-1α axis	Oncogene	Promotes the proliferation, migration, invasion and glycolysis of GC cells. Promotes malignant behaviors and metabolic reprogramming of GC	Li et al. (2022)
circBFAR	*In vitro* and *in vivo*	Up	miR-513a-3p/HK2 axis	Oncogene	Promotes proliferation and glycolysis in GC	Wang et al. (2021b)

Abbreviations: THUMPD3-AS1, THUMPD3 antisense RNA, one; LINC00242, Long intergenic non-protein coding RNA, 242; circDNMT1, circRNA DNA, methyltransferase one; circBFAR, circular RNA, bifunctional apoptosis regulator (circBFAR); PDCD4, Programmed cell death protein 4; NUAK1, Novel (nua) kinase family one; KDM2B, Human Lysine-specific demethylase 2B; CXCL17, Chemokine (C-X-C motif) ligand 17; G6PD, Glucose-6-phosphate dehydrogenase; LDHA, Lactate dehydrogenase A; MYH9, Myosin heavy chain nine; HIF-1α, Hypoxia-inducible factor 1-alpha.

## 8 Clinical perspective of ncRNAs in GC radiotherapy

**FIGURE 4:**
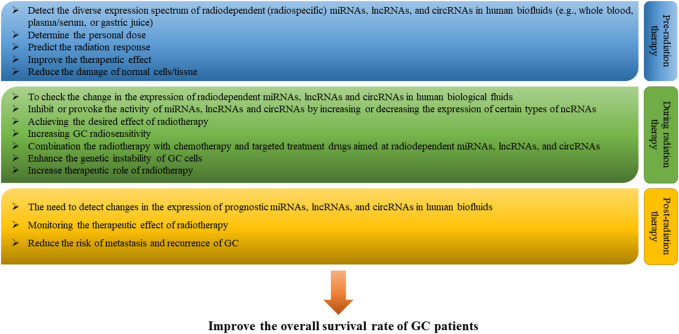
Clinical perspective of microRNAs (miRNAs), long non-coding RNAs (lncRNAs) and circular RNAs (circRNAs) in gastric cancer (GC) radiotherapy. In the future, in clinical practice with the use of radiotherapy, it is possible to assess and monitor the range of changes in the expression of radiodependent miRNAs, lncRNAs, and circRNAs in human biofluids (e.g., whole blood, plasma/serum, or gastric juice) pre-radiation therapy, during radiation therapy, and post-radiation therapy periods in order to enhance the therapeutic effect of radiotherapy and improve the overall survival of GC patients.

There are suggestions that the direct involvement of miRNAs, lncRNAs, and circRNAs in GC radiosensitivity is likely to be applied in clinical practice in the near future. And this possibility may involve many steps. First, before radiotherapy is given to patients with GC, it will be necessary to assess the range of expression changes in radiodependent miRNAs, lncRNAs, and circRNAs in human biofluids (e.g., whole blood, plasma/serum, or gastric juice), to in order to: 1) predict the response to radiation of each specific patient, 2) determine the individual radiation dose, and 3) minimize acute and latent damage to normal cells/tissues (Das et al., 2019; May et al., 2021). Secondly, in the course of radiotherapy, checking the expression change in radiodependent miRNAs, lncRNAs, and circRNAs in biological fluids and changing the expression of a number of certain miRNAs, lncRNAs, and circRNAs among radiodependent ncRNAs can help to effectively achieve the desired effect of radiation therapy and further increase radiosensitivity of GC. Thirdly, during the period of radiotherapy, radiation therapy itself can be combined with chemotherapy drugs (e.g., oxaliplatin (FLO) or cisplatin (FLP)), small molecule inhibitors (e.g., tyrosine kinase inhibitors) and drugs that target specific miRNAs, lncRNAs, and circRNAs to enhance the genetic instability of cancer cells, increase the rate of destruction of radiation, and enhance the overall effect of radiotherapy (Song et al., 2017; Patel and Cecchini, 2020; Fong et al., 2022). And fourth, when radiotherapy is completed, determining the expression of so-called predictive miRNAs, lncRNAs, and circRNAs in body fluids can help control the therapeutic effect of radiation and reduce the risk of metastasis and recurrence of GC (Figure 4) (Wei et al., 2020). In addition, the discovery of the role of miRNAs, lncRNAs, and circRNAs, as well as their interaction with each other, in the regulation of GC radiosensitivity increases the likelihood that these ncRNAs, in particular radio-dependent ones, will provide a promising direction in the clinical practice of prevention, diagnosis, prognosis and treatment of GC.

Advances in fluid biopsy, that is, the non-invasive detection of radiospecific miRNAs, lncRNAs and circRNAs as biomarkers in biological fluids to assess response to GC radiotherapy, are entirely possible. In addition, the potential to improve the radiotherapeutic effect by activating or inhibiting the expression of certain miRNAs, lncRNAs and circRNAs and downstream target genes is extremely promising.

## 9 Conclusion

To sum up, ncRNAs may play a role in regulating the sensitivity of GC to radiation therapy by impacting important biological processes such as DNA damage response, cell stemness, apoptosis, EGFR activation, EMT, and oxygen adaptive metabolism. This suggests new opportunities for research to further investigate the impact of ncRNA dysregulation on the radiation sensitivity of GC. Further studies are needed to explore the potential role of ncRNA in regulating GC radiation sensitivity in-depth, and to develop a prediction model and screening system for ncRNAs to regulate radiation sensitivity of GC, which can bring new hope to improve the prognosis of GC patients. Overall, the role of ncRNA in the development and progression of tumors is a current area of focus in tumor biology research. Targeting ncRNA may be an effective method to reduce the resistance of GC to radiation therapy, which can help improve the effectiveness of radiation therapy for GC patients and provide new ideas and strategies for GC radiation therapy.
